# Pain Perception in Unresponsive Wakefulness Syndrome May Challenge the Interruption of Artificial Nutrition and Hydration: Neuroethics in Action

**DOI:** 10.3389/fneur.2016.00202

**Published:** 2016-11-16

**Authors:** Francesco Riganello, Simone Macrì, Enrico Alleva, Carlo Petrini, Andrea Soddu, Josè Leòn-Carriòn, Giuliano Dolce

**Affiliations:** ^1^Research in Advanced Neurorehabilitation, Istituto S. Anna, Crotone, Italy; ^2^Section of Behavioral Neuroscience, Department of Cell Biology and Neuroscience, Istituto Superiore di Sanità, Rome, Italy; ^3^Office of the President, Bioethics Unit, Istituto Superiore di Sanità, Rome, Italy; ^4^Department of Physics and Astronomy, Brain and Mind Institute, The University of Western Ontario, London, ON, Canada; ^5^Human Neuropsychology Laboratory, Department of Experimental Psychology, School of Psychology, University of Seville, Seville, Spain

**Keywords:** unresponsive wakefulness syndrome, vegetative state, pain, consciousness, withdrawal of artificial nutrition and hydration, neuroethics

## Introduction

The voluntary withdrawal of artificial nutrition and hydration (ANH) in patients with severe disorders of consciousness (DoC, e.g., permanent vegetative states) constitutes a fundamental ethical issue extending beyond the boundaries of end-of-life decisions. The term “Vegetative State” was originally adopted to define “an organic body capable of growth and development but devoid of sensation and thought” (1). In this frame, the traditional approach toward the suspension of ANH rested upon the view that patients in the Vegetative State had no residual capability to perceive pain (2, 3). However, over the past few decades, an increasing number of studies (4–11) have shown that strong claims about awareness in patients without behavioral responses to commands are unwarranted. Furthermore, there is again a high percentage of misdiagnoses in the assessment of these patients (12–14).

Recently it was proposed the alternative name “unresponsive wakefulness syndrome” (VS/UWS) (15), to define a condition characterized by the absence of response to commands or oriented voluntary movements in the presence of wakefulness. Neuroimaging studies demonstrated the existence of distinctive cerebral responses to noxious stimuli in conditions like VS/UWS and minimally conscious state (MCS) (16–18). Yet, there is no univocal consensus about pain perception in patients with DoC. In a study by Demertzi and colleagues (19), 2,059 medical and paramedical professionals from 32 European countries were asked the following question: “can a patient in vegetative state feel pain?” Over 40% of those surveyed replied that these patients do not feel pain. The percentage was higher among medical doctors compared to paramedical caregivers (54 and 32%, respectively). This view was substantiated by the idea that, in the absence of consciousness, patients were not capable of experiencing and reporting a painful experience. However, recent advances in the pathophysiology of DoC, debunking the original tenet that patients with DoC do not perceive pain, suggest a reconsideration of the voluntary withdrawal of ANH (20).

In this manuscript, we will briefly review the recent literature indicating that some patients with DoC reveal a form of residual awareness (21) and that they are capable of perceiving painful stimuli and exhibiting consistent responses to them. Furthermore, empirical evidence in the literature suggests that, when tested with the appropriate tools, these patients can exhibit consistent reactions to emotionally salient stimuli (5, 10, 22–27). Based on these findings, we propose that the voluntary withdrawal of ANH should be carefully reconsidered on medical and ethical grounding.

## Pain: Definition and Neuroanatomical Substrates

Pain is defined as “… an unpleasant sensory and emotional experience arising from actual or potential tissue damage or described in terms of such damage…” (28). Likewise, McCaffrey and Pasero (29) reported that “Pain is *whatever the experiencing person says it is, existing whenever* the experiencing person *says it does*.” Such subjectivity implies that pain may only be detected when a patient reports its manifestation. Therefore, the presence of consciousness would seem to constitute a fundamental prerequisite in the individual appraisal and experience of pain. Analogous considerations may be applied to the concept of suffering, defined as “the state of severe distress associated with events that threaten the intactness of the person” (30). Furthermore, Turk and Wilson (31) proposed that “a person might experience significant pain-related suffering from a relatively low-level noxious stimulation if she or he believes the implications are ominous, interminable, and beyond their control” (31). Within this framework, both pain and suffering seem to require the presence of consciousness.

Several authors have shown that nociceptive stimuli elicit the activation of an extensive cortical network including somatosensory, insular, and cingulate areas, as well as frontal and parietal areas (32, 33). In particular, nociception refers to the perception (conscious or not) of nociceptive stimuli (28). The stimulation of nociceptors leads to the transmission of information *via* the spinothalamic tract to the thalamus and on to the cortex, with the midbrain and thalamus thought to be involved in the modulation of reflex response to nociceptive stimuli (34). The cortical nociceptive network entails the secondary somatosensory (S2) cortex, with the posterior insula (lateral network), taking part in the sensory–discriminative features of the pain processing (35, 36). However, the generation of a conscious experience of pain requires the activation of a more complex network. This network is often referred to as the “pain matrix.” The pain matrix entails two main subsystems (37): the lateral neuronal network [secondary somatosensory (S2) cortex, lateral thalamus, and posterior insula], encoding sensory–discriminative information (38), and the medial network [anterior cingulate cortex (ACC) and prefrontal cortex], encoding affective–cognitive information (39). Also, the cerebellum plays a major role in processing aversive stimuli including pain (40), contributing to the sensory–discriminative part of the pain processing network. Finally, motor-related areas (e.g., the striatum, cerebellum, and the supplementary motor area) are involved in pain perception and processing (41). In addition to identifying the neuroanatomical structures involved in pain perception, these studies investigated the dynamics of activation (connectivity) of the pain matrix. Thus, results on cortical potential suggest that nociceptive input is first processed in the posterior insula, wherein it is coded in terms of intensity and anatomical location, and then conveyed to the anterior insula, where the emotional reaction to pain is elaborated (36, 42, 43).

Furthermore, several authors demonstrated the existence in humans of multiple somatotopic representations of pain within the operculo-insular region. This mechanism may constitute a sensory-integration site that selectively targets emotional responses toward the specific injured body-site (44). Finally, the pain matrix has been shown to vary with age and in response to the onset of pathologies. Some authors have suggested that structural deficits in regions involved in the modulation of pain, and in their connectivity, occur in chronic pain syndromes (45).

## Pain in Patients with DOC

Pioneering studies attempting to quantify nociception in patients with DoC (46) systematically measured physiological variables like eye opening, breathing, heart rate, and blood pressure, and occasionally grimace-like or crying-like behavior (47). However, notwithstanding their heuristic value, these signs are considered to be of subcortical origin, not necessarily reflecting a conscious perception of pain. Additional quantitative measurements of nociception in patients with DoC reported stereotyped responses (i.e., slow generalized flexion or extension of the upper and lower extremities), flexion withdrawal (i.e., withdrawal of the limb away from the point of the stimulation), and localization responses (i.e., the non-stimulated limb locates and makes contact with the stimulated body part at the point of stimulation), all of which were linked, respectively, to brainstem, subcortical, or cortical activity (nociceptive network or pain matrix) (32, 46, 48).

Other studies investigated the extent to which the pain matrix responded to nociceptive stimuli in patients with DoC. For example, Boly et al. (16) observed that, in response to a noxious stimulus, patients in MCS showed “pain matrix” activation similar to that seen in normal controls (16). Patients in VS/UWS also showed activation in the pain matrix, although this activation was much less prominent compared to controls (16). An independent study performed by Markl et al. (49) observed the presence of significant activation in the sensory and affective components of the pain matrix in patients characterized by severe DoC (49). In a study adopting laser-evoked potentials, the authors observed that painful stimuli may be processed even in patients with severe brain damage (50). Functional magnetic resonance imaging (fMRI) has also been used to investigate brain mechanisms underlying the perception of pain in patients with DoC. Using this paradigm, Monti et al. (20) demonstrated that patients with a diagnosis of “unresponsive wakefulness syndrome” (UWS), evaluated at the bedside, exhibited measurable awareness. Therefore, the authors proposed that negative results in clinical trials or activation protocols do not necessarily imply the absence of cognitive processes in these patients (20). Alternatively, it may be suggested that these patients were misdiagnosed due to the technical constraints of the bedside evaluation. Retrospective reports of patients surviving intensive care constitute an additional indication that patients with DoC may exhibit some form of appraisal of noxious stimuli. These individuals report vivid memories of pain, noise, sleep deprivation, thirst, hunger, heat, cold, fear, anxiety, isolation, physical restraint, lack of information, and absence of daylight (51).

These empirical data corroborate the view that although patients with DoC may not be capable of exhibiting a detectable reaction to painful stimuli, they may nonetheless be capable of perceiving them (52). These data do not demonstrate that all patients with severe disorders of consciousness perceive and/or exhibit pain; yet, they demonstrate that several patients, deemed incapable of experiencing pain, showed a consistent response to noxious stimuli. Regardless of whether this is due to misdiagnosis or technical limitations, these data contravene the tenet that all patients with severe disorders of consciousness do not experience pain.

## Conclusion

For patients with severe pathologies (e.g., terminally ill), the dehydration and starvation may have benefits (patients could be intolerant of enteral feeding because of abdominal distension, vomiting, diarrhea, or fluid overload) and to refuse food and fluids and to have relief of distress through provision of medicine may be a right (53). However, most patients in VS/UWS are unlikely to be intolerant of nutrition and hydration that are considered basic compassionate care because they promote physical and emotional well-being (54, 55). Withdrawal of ANH has biologic consequences including distress and pain (56).

Neurophysiological and fMRI studies are finding ways to assess awareness in VS/UWS patients (57, 58). The identification of the “pain matrix” along with the design of experimental tools capable of detecting consistent patterns of brain activation in response to noxious stimuli allowed us to integrate the original definitions of pain, which rested largely upon subjective experiences with objective measurements.

These data, which bestow a heuristic advancement in the study of pain and consciousness, may also foster a reconsideration of the general attitude toward the management of pain in patients with DoC. Herein, our main interest is directed toward the withdrawal of ANH in these patients. With respect to the latter, Ogino and collaborators (59) demonstrated that dehydration, in control subjects, leads to increased brain activity in the anatomical structures involved in pain perception (ACC, insula, and thalamus). Thus, dehydration may *per se* constitute a source of pain in healthy subjects (60). Furthermore, Perry et al. (60) reported that hydration status is an important modulator of the cerebrovascular response to cold pressor tests, which are commonly used to investigate cerebrovascular regulation. Therefore, dehydration may not only constitute a source of pain but also interfere with the ability to assess a nociceptive response in the patient. Ultimately, current experimental knowledge supports the notion that voluntary withdrawal of hydration may, under specific conditions, constitute a source of pain. Given the empirical data reported above, concerning pain perception in patients with DoC, it is tenable that the primary ethical, clinical, and deontological concerns shall relate to the suffering of the patient. Within this framework, the voluntary withdrawal of ANH may raise ethical concerns worth being addressed.

## Author Contributions

All the authors have contributed to the drafting and revisions of the manuscript and have approved the submitted version.

## Note

From the meeting “Voluntary withdrawal of Nutrition and Hydration in Patients with severe Disorders of Consciousness: Clinical and Ethical aspects.” 23-11-2015 Rome (Italy).

## Conflict of Interest Statement

The authors declare that the research was conducted in the absence of any commercial or financial relationships that could be construed as a potential conflict of interest.

## References

[1] Jennett B Plum F Persistent vegetative state after brain damage. A syndrome in search of a name. Lancet Lond Engl (1972) 1:734–7. 10.1016/S0140-6736(72)90242-5 4111204

[2] Andrews K. Medical decision making in the vegetative state: withdrawal of nutrition and hydration. NeuroRehabilitation (2004) 19:299–304. 15671584

[3] Jennett B Thirty years of the vegetative state: clinical, ethical and legal problems. In: Laureys S, editor. Progress in Brain Research. The Boundaries of Consciousness: Neurobiology and Neuropathology. Elsevier (2005). p. 537–43. Available from: http://www.sciencedirect.com/science/article/pii/S0079612305500372 10.1016/S0079-6123(05)50037-2 16186047

[4] Bruno MA Vanhaudenhuyse A Schnakers C Boly M Gosseries O Demertzi A Visual fixation in the vegetative state: an observational case series PET study. BMC Neurol (2010) 10:35. 10.1186/1471-2377-10-35 20504324 PMC2895583

[5] Dolce G Riganello F Quintieri M Candelieri A Conforti D Personal interaction in the vegetative state: a data-mining study. J Psychophysiol (2008) 22:150–6. 10.1027/0269-8803.22.3.150

[6] Gutiérrez J Machado C Estévez M Olivares A Hernández H Perez J Heart rate variability changes induced by auditory stimulation in persistent vegetative state. Int J Disabil Hum Dev (2010) 9:357–62. 10.1515/IJDHD.2010.041

[7] Laureys S Perrin F Brédart S. Self-consciousness in non-communicative patients. Conscious Cogn (2007) 16:722–41. 10.1016/j.concog.2007.04.004 17544299

[8] Leon-Carrion J Leon-Dominguez U Halper J Pollonini L Zouridakis G Dominguez-Morales M Restoring cortical connectivity directionality and synchronization is essential to treating disorder of consciousness. Curr Pharm Des (2013) 999:17–8. 10.2174/13816128113196660651 24025059

[9] Monti MM. Cognition in the vegetative state. Annu Rev Clin Psychol (2012) 8:431–54. 10.1146/annurev-clinpsy-032511-143050 22224835

[10] Riganello F Candelieri A Dolce G Sannita WG Residual emotional processing in the vegetative state: a scientific issue? Clin Neurophysiol (2011) 122:1061–2. 10.1016/j.clinph.2010.09.006

[11] Riganello F Cortese MD Dolce G Sannita WG. Visual pursuit response in the severe disorder of consciousness: modulation by the central autonomic system and a predictive model. BMC Neurol (2013) 13:164. 10.1186/1471-2377-13-164 24195685 PMC3832247

[12] Andrews K Murphy L Munday R Littlewood C. Misdiagnosis of the vegetative state: retrospective study in a rehabilitation unit. BMJ (1996) 313:13–6. 10.1136/bmj.313.7048.13 8664760 PMC2351462

[13] Bosco A Lancioni GE Belardinelli MO Singh NN O’Reilly MF Sigafoos J. Vegetative state: efforts to curb misdiagnosis. Cogn Process (2010) 11:87–90. 10.1007/s10339-009-0355-y 20043186

[14] Gill-Thwaites H. Lotteries, loopholes and luck: misdiagnosis in the vegetative state patient. Brain Inj (2006) 20:1321–8. 10.1080/02699050601081802 17378223

[15] Laureys S Celesia GG Cohadon F Lavrijsen J León-Carrión J Sannita WG Unresponsive wakefulness syndrome: a new name for the vegetative state or apallic syndrome. BMC Med (2010) 8:68. 10.1186/1741-7015-8-68 21040571 PMC2987895

[16] Boly M Faymonville ME Schnakers C Peigneux P Lambermont B Phillips C Perception of pain in the minimally conscious state with PET activation: an observational study. Lancet Neurol (2008) 7:1013–20. 10.1016/S1474-4422(08)70219-9 18835749

[17] Laureys S Death, unconsciousness and the brain. Nat Rev Neurosci (2005) 6:899–909. 10.1038/nrn1789 16261182

[18] Laureys S Faymonville ME Peigneux P Damas P Lambermont B Del Fiore G Cortical processing of noxious somatosensory stimuli in the persistent vegetative state. Neuroimage (2002) 17:732–41. 10.1006/nimg.2002.1236 12377148

[19] Demertzi A Schnakers C Ledoux D Chatelle C Bruno MA Vanhaudenhuyse A Different beliefs about pain perception in the vegetative and minimally conscious states: a European survey of medical and paramedical professionals. Progress in Brain Research. Elsevier (2009). p. 329–38. Available from: http://linkinghub.elsevier.com/retrieve/pii/S0079612309177221 10.1016/S0079-6123(09)17722-1 19818911

[20] Monti MM Laureys S Owen AM The vegetative state. BMJ (2010) 341:c3765–3765. 10.1136/bmj.c3765 20679291

[21] Chennu S Finoia P Kamau E Allanson J Williams GB Monti MM Spectral signatures of reorganised brain networks in disorders of consciousness. PLoS Comput Biol (2014) 10:e1003887. 10.1371/journal.pcbi.1003887 25329398 PMC4199497

[22] Bekinschtein T Niklison J Sigman L Manes F Leiguarda R Armony J Emotion processing in the minimally conscious state. J Neurol Neurosurg Psychiatry (2004) 75:788–788. 10.1136/jnnp.2003.034876 15090585 PMC1763566

[23] Fiacconi CM Owen AM Using Facial Electromyography to Detect Preserved Emotional Processing in the Vegetative State in Psychophysiology. Hoboken, NJ: Wiley-Blackwell (2015). p. S35–35.

[24] Machado C Korein J Aubert E Bosch J Alvarez MA Rodríguez R Recognizing a mother’s voice in the persistent vegetative state. Clin EEG Neurosci (2007) 38:124–6. 10.1177/155005940703800306 17844939

[25] O’Kelly J James L Palaniappan R Taborin J Fachner J Magee WL. Neurophysiological and behavioral responses to music therapy in vegetative and minimally conscious states. Front Hum Neurosci (2013) 7:884. 10.3389/fnhum.2013.00884 24399950 PMC3872324

[26] Riganello F Candelieri A Quintieri M Conforti D Dolce G. Heart rate variability: an index of brain processing in vegetative state? An artificial intelligence, data mining study. Clin Neurophysiol (2010) 121:2024–34. 10.1016/j.clinph.2010.05.010 20566300

[27] Sharon H Pasternak Y Ben Simon E Gruberger M Giladi N Krimchanski BZ Emotional processing of personally familiar faces in the vegetative state. PLoS One (2013) 8:e74711. 10.1371/journal.pone.0074711 24086365 PMC3783455

[28] IASP. Classification of Chronic Pain: Descriptions of Chronic Pain Syndromes and Definitions of Pain Terms. Seattle, WA: IASP Press (1994).

[29] McCaffrey M Pasero C Pain: Clinical manual. St Louis, MO: Mosby (1999).

[30] Cassell EJ Recognizing suffering. Hastings Cent Rep (1991) 21:24–24. 10.2307/3563319 1885294

[31] Turk DC Wilson HD Pain, suffering, pain-related suffering – are these constructs inextricably linked? Clin J Pain (2009) 25:353–5. 10.1097/AJP.0b013e31819c62e7 19454867

[32] Chatelle C Thibaut A Bruno MA Boly M Bernard C Hustinx R Nociception coma scale-revised scores correlate with metabolism in the anterior cingulate cortex. J Neurol Rehabil (2014) 28:149–52. 10.1177/1545968313503220 24065132

[33] Coghill RC McHaffie JG Yen Y-F. Neural correlates of interindividual differences in the subjective experience of pain. Proc Natl Acad Sci U S A (2003) 100:8538–42. 10.1073/pnas.1430684100 12824463 PMC166264

[34] Loeser JD Treede R-D The Kyoto protocol of IASP basic pain terminology. Pain (2008) 137:473–7. 10.1016/j.pain.2008.04.025 18583048

[35] Lockwood PL Iannetti GD Haggard P. Transcranial magnetic stimulation over human secondary somatosensory cortex disrupts perception of pain intensity. Cortex (2013) 49:2201–9. 10.1016/j.cortex.2012.10.006 23290634 PMC4412907

[36] Ploner M Gross J Timmermann L Schnitzler A. Cortical representation of first and second pain sensation in humans. Proc Natl Acad Sci U S A (2002) 99:12444–8. 10.1073/pnas.182272899 12209003 PMC129464

[37] Brooks J Tracey I. From nociception to pain perception: imaging the spinal and supraspinal pathways. J Anat (2005) 207:19. 10.1111/j.1469-7580.2005.00428.x 16011543 PMC1571498

[38] Mutschler I Wankerl J Seifritz E Ball T P02-405 – the role of the human insular cortex in pain processing. Eur Psychiatry (2011) 26(Suppl 1):1001 10.1016/S0924-9338(11)72706-7

[39] Medford N Critchley HD. Conjoint activity of anterior insular and anterior cingulate cortex: awareness and response. Brain Struct Funct (2010) 214:535–49. 10.1007/s00429-010-0265-x 20512367 PMC2886906

[40] Moulton EA Elman I Pendse G Schmahmann J Becerra L Borsook D. Aversion-related circuitry in the cerebellum: responses to noxious heat and unpleasant images. J Neurosci (2011) 31:3795–804. 10.1523/JNEUROSCI.6709-10.2011 21389234 PMC3063442

[41] Barceló AC Filippini B Pazo JH. The striatum and pain modulation. Cell Mol Neurobiol (2012) 32:1–12. 10.1007/s10571-011-9737-7 21789630 PMC11498585

[42] Frot M Faillenot I Mauguière F. Processing of nociceptive input from posterior to anterior insula in humans. Hum Brain Mapp (2014) 35:5486–99. 10.1002/hbm.22565 24916602 PMC6869247

[43] Tracey I. Imaging pain. Br J Anaesth (2008) 101:32–9. 10.1093/bja/aen102 18556697

[44] Baumgärtner U Iannetti GD Zambreanu L Stoeter P Treede R-D Tracey I. Multiple somatotopic representations of heat and mechanical pain in the operculo-insular cortex: a high-resolution fMRI study. J Neurophysiol (2010) 104:2863–72. 10.1152/jn.00253.2010 20739597 PMC2997041

[45] Hadjipavlou G Dunckley P Behrens TE Tracey I. Determining anatomical connectivities between cortical and brainstem pain processing regions in humans: a diffusion tensor imaging study in healthy controls. Pain (2006) 123:169–78. 10.1016/j.pain.2006.02.027 16616418

[46] Schnakers C Chatelle C Vanhaudenhuyse A Majerus S Ledoux D Boly M The Nociception Coma Scale: a new tool to assess nociception in disorders of consciousness. Pain (2010) 148:215–9. 10.1016/j.pain.2009.09.028 19854576

[47] Schnakers C Zasler ND. Pain assessment and management in disorders of consciousness. Curr Opin Neurol (2007) 20:620–6. 10.1097/WCO.0b013e3282f169d9 17992079

[48] Riganello F Cortese MD Arcuri F Candelieri A Guglielmino F Dolce G A study of the reliability of the Nociception Coma Scale. Clin Rehabil (2014) 29:388–93. 10.1177/0269215514546767 25172088

[49] Markl A Yu T Vogel D Müller F Kotchoubey B Lang S. Brain processing of pain in patients with unresponsive wakefulness syndrome. Brain Behav (2013) 3:95–103. 10.1002/brb3.110 23533065 PMC3607151

[50] de Tommaso M Navarro J Lanzillotti C Ricci K Buonocunto F Livrea P Cortical responses to salient nociceptive and not nociceptive stimuli in vegetative and minimal conscious state. Front Hum Neurosci (2015) 9:17 10.3389/fnhum.2015.00017 25688200 PMC4310288

[51] Simini B Patients’ perceptions of intensive care. Lancet (1999) 354:571–2. 10.1016/S0140-6736(99)02728-2 10470711

[52] Naro A Leo A Bramanti P Calabrò RS. Moving toward conscious pain processing detection in chronic disorders of consciousness: anterior cingulate cortex neuromodulation. J Pain (2015) 16:1022–31. 10.1016/j.jpain.2015.06.014 26208761

[53] Savulescu J A simple solution to the puzzles of end of life? Voluntary palliated starvation. J Med Ethics (2013) 2013:101379 10.1136/medethics-2013-101379 23869048

[54] Amano K Morita T Baba M Kawasaki M Nakajima S Uemura M Effect of nutritional support on terminally ill patients with cancer in a palliative care unit. Am J Hosp Palliat Care (2013) 30:730–3. 10.1177/1049909112469273 23242171

[55] Cohen MZ Torres-Vigil I Burbach BE de la Rosa A Bruera E. The meaning of parenteral hydration to family caregivers and patients with advanced cancer receiving hospice care. J Pain Symptom Manage (2012) 43:855–65. 10.1016/j.jpainsymman.2011.06.016 22459230 PMC3354988

[56] Rady MY Verheijde JL. Nonconsensual withdrawal of nutrition and hydration in prolonged disorders of consciousness: authoritarianism and trustworthiness in medicine. Philos Ethics Humanit Med (2014) 9:16. 10.1186/1747-5341-9-16 25381041 PMC4304039

[57] Gosseries O Di H Laureys S Boly M. Measuring consciousness in severely damaged brains. Annu Rev Neurosci (2014) 37:457–78. 10.1146/annurev-neuro-062012-170339 25002279

[58] Jox RJ Bernat JL Laureys S Racine E. Disorders of consciousness: responding to requests for novel diagnostic and therapeutic interventions. Lancet Neurol (2012) 11:732–8. 10.1016/S1474-4422(12)70154-0 22814543

[59] Ogino Y Kakeda T Nakamura K Saito S. Dehydration enhances pain-evoked activation in the human brain compared with rehydration. Anesth Analg (2014) 118:1317–25. 10.1213/ANE.0b013e3182a9b028 24384865

[60] Perry BG Bear TLK Lucas SJE Mündel T Mild dehydration modifies the cerebrovascular response to the cold pressor test: hydration status and the cold pressor test. Exp Physiol (2016) 101(1):135–42. 10.1113/EP085449 26374269

